# Effect of fins in enhancing phase change material fusion in a spherical thermal energy storage container

**DOI:** 10.1038/s41598-026-38262-8

**Published:** 2026-02-12

**Authors:** Banumathi Munuswamy Swami Punniakodi, M. Veeramanikandan, M. Manickam, A. Arunkumar, Dhinesh Balasubramanian, Utku Kale, Artūras Kilikevičius, Vilma Locaitienė

**Affiliations:** 1 SD Industrial Works, Chennai, Tamil Nadu , India; 2 Department of Mechanical Engineering, Faculty of Engineering and Technology, SRM Institute of Science and Technology, Ramapuram Campus, Chennai, Tamil Nadu India; 3 Department of Mechanical Engineering, Dhanalakshmi Srinivasan University, Samayapuram, Tiruchirapalli, Tamil Nadu India; 4 Department of Mechanical Engineering, E.G.S. Pillay Engineering College, Nagapattinam, Tamilnadu India; 5 Department of Port Engineering, Vilnius Gediminas Technical University, Lithuanian Maritime Academy (LMA), Klaipėda, Lithuania; 6 Department of Aeronautics and Naval Architecture, Faculty of Transportation Engineering and Vehicle Engineering, Budapest University of Technology and Economics, Műegyetem rkp. 3., Budapest, H-1111 Hungary; 7 Mechanical Science Institute, Vilnius Gediminas Technical University, Plytinės g. 25, LT-10105 Vilnius, Lithuania

**Keywords:** Finned spherical container, Thermal energy storage, Phase change material, Heat storage/release, Heat transfer fluid, Energy science and technology, Engineering, Materials science

## Abstract

The increased depletion of fossil fuels needs alternatives like solar energy to address daily demands. However, solar energy is intermittent, hence it is integrated with thermal energy storage (TES) systems. Phase change materials (PCM) are used in TES systems, but they suffer from meagre thermal conductivity, leading to less fusion. This study enhances PCM fusion using four different cases, namely, case 1 (without fins), case 2 (with two fins placed near the top), case 3 (with two fins placed near the bottom), and case 4 (with two fins placed near the top and bottom), respectively. The study is done with heat transfer fluid (HTF) temperature at 70 °C and 75 °C with a 30 LPH flow rate. Case 4 displayed a melting time reduction of 34%, and 47.7% when compared with case 1. It is also found with high thermal efficiency and effectiveness. The results indicate that case 4 is effective in expediting fusion and enhancing storage performance due to optimal fin placement, hence suggested for solar applications.

## Introduction

The fossil fuels increased use led to pollution and climate change. It also led to depletion, hence an alternative like solar energy is opted. The renewable energy, like solar, is abundant, but its intermittent nature needs more storage during availability. Hence, thermal energy storage (TES) systems are opted. TES happens at close isothermal conditions during phase change. The phase change heat is high; hence, phase change materials (PCM) are opted for energy storage/release. However, it suffers from meagre thermal conductivity. Studies are done to address it using nanoparticles^1,2^, fins^3,4^, heat transfer tubes^5,6^, containers^7^ and metal mesh^8^, respectively. The combined fins and nanoparticles also increase heat storage^9^ along with honeycomb-based structures^10^. Further TES find application in solar PV^11,12^, collectors^13^, trombe wall^14^, and heat recovery in diesel engines^15^.

Goud Ranga et al.^16^ analysed PCM melting using nanoparticles like Cu, Al_2_O_3,_ graphene and CuO, at concentrations of 2, 5, 8, and 10%. The 10% nanoparticle concentration without fins enhanced PCM melting and reduced the fusion time to 1180 s from 3000 s. Rashid et al.^17^ analysed PCM solidification. Three cases are analysed, namely, fins, nanoparticles, and fins along with nanoparticles in a container. He et al.^18^ analysed PCM melting using heat transfer oil. The melting is found with different stages, namely, channel forming, melting and end melting stage. During the initial stage, conduction was dominant, and it is followed by convection in the next stage. The final stage is with sensible heat accumulation.

Soliman et al.^19^ analysed PCM melting using convex/concave dimples on the walls, nanoparticles, and heat flux. Six dimples reduced the melt time by 15.56% than smooth wall. Further, the capsule incorporating PCM influences melting^20^. Sattinova et al.^21^ analysed melting using fins and beryllium oxide nanoparticle (1, 3, and 5%). PCM thermal conductivity is enhanced by 15% at 5% nanoparticle concentration. Fin numbers, namely, 2, 4, and 6, reduce melt time by 30.6, 44.4, and 52.8% compared to the base case. Nanoparticle addition at 1, 3, and 5% reduced melt time by 2.8, 7.2, and 12.2%. Hajizadeh et al.^22^ analysed PCM fusion using fins and nanoparticles. Three cases were studied to enhance fusion, namely, no fin and two fin cases with different arrangements. Nanoparticle inclusion with fins reduced the fusion time from 19.16 to 5.83 min. Kok et al.^23^ analysed nano-PCM and fins in enhancing melting. Different fins are used to enhance melting. The melt time is reduced by 63% using optimal fins.

Li et al.^24^ analysed PCM fusion using fins and nanoparticles. Three loading of graphene nanoplatelets (GNP), and different fins, namely, helical, annular, and longitudinal, were used for the analysis. The optimal fin and GNP loading enhance heat transport. The longitudinal fin with GNP is found to have a negative impact on enhancing heat storage. Singh et al.^25^ analysed PCM melting using various GNP proportions and fins. The melting time is reduced by 60% in a finned TES with 5% GNP compared to a conventional system. Ul Hasnain et al.^26^ enhanced PCM melting using branched fins and nanoparticles. Fin geometry like straight, single-branched, and double-branched reduces melt time by 11.5, 19.2, and 26.8%, compared with the Y-shaped triple fin. Nanoparticle addition of 1, 5, and 10% results in melt time savings (11.5, 19.2, and 26.8%), compared with PCM.

Bellan et al.^27^ analysed salt PCMs for storing energy. The corrosivity of salts is reduced using ceramic capsules. Increasing Stefan number (0.089 to 0.282) increases melting and reduces fusion time by 64%. Jaberi and Hossainpour^28^ analysed PCM melting using porous fins. The melting time reduction of 21% and increase in storage energy of 26. 6% is achieved using porous fins at lower locations. Shaker et al.^29^ analysed the fusion process of cylindrical encapsulated PCM. Parameters, namely, heat transfer fluid (HTF) temperature, PCM, flow rate, solidification temperature and fin number were analysed. Flow rate rise by five times reduces lauric acid and wax melt time by 30 and 22%. Fins’ presence enhances fusion.

Hosseini et al.^30^ analysed PCM melting using annular and longitudinal fins. The TES with longitudinal fins is more optimal than annular fins. PCM melted at 17% earlier using longitudinal fins than annular fins. Piran et al.^31^ analysed a twisted tube along with spiral V-shaped axial fins to enhance fusion. Parameters, namely, pitch, number, and fin height, are analysed. Fins reduced melt time by 32.29, 52.08, 59.38, and 65.42% compared with the finless case when their numbers changed from one to four.

Troxler et al.^32^ analysed melting in a rectangular container (50 mm × 50 mm × 120 mm) subjected to an isothermal conduction at angles ranging from 30 to 90° from horizontal. The melting speed is enhanced by 35% for 47°, and it is 46% for 57°. Wang et al.^33^ studied PCM charging using three different fins in TES, namely, A, B, and C; horizontal fins, vertical transverse, and vertical longitudinal fins. Melting rate is enhanced for A (4.52 times), B (3.63 times), and C (3.62 times) compared with the finless case. Deng et al.^34^ analysed PCM melting using a double fin at different inclination angles (30, 60, 90, 120, 150, and 180°). Melt time is reduced by 66.7% when the dimensionless fin length increases from 0.5 to 1. Patel et al.^35^ analysed PCM fusion using different TES capsules and their geometry. Triangular TES is found optimal with a melting and solidification time of 41 and 133 min, respectively. The capsule size reduction of 27% reduces the melting and solidification time by 12.19 and 19.17% indicating that capsule size affects PCM fusion.

Jaffray et al.^36^ analysed PCM melting and solidification. Natural convection was dominant during melting and conduction during solidification. Zhu et al.^37^ analysed cylindrical TES using different fins. With a heat flux of 800 W. m^−2^ for the finless case, the PCM melt at 2813 s. The Tra-45 fin reduced melt time by 5.4% compared with the finless case. Yu et al.^38^ analysed molten salt melting in a TES container. Parameters like liquid fraction, heat transfer dimensionless parameter, temperature, and storage were analysed. High heating temperature and a large aspect ratio enhance melting and storage. Du et al.^39^ analysed the flow rate for optimal energy storage/release. Increasing flow rate (from 5 to 10 L. min^−1^) varies the phase change. Increasing the pipe to two reduces phase change time by 70.3% and 67.1% for 5 and 10 l. min^−1^. Increasing flow rate reduces average temperature uniformity.

Sathishkumar et al.^40^ analysed the fusion of water with graphene nanoplatelets (0.3, 0.6, 0.9, and 1.2%) in a spherical container. The addition of nanoparticles reduced the subcooling of water from − 7 to − 2.5 °C, resulting in a 25% reduction in fusion time. Hekmat and Saharkhiz^41^ used helical shell and tube TES to enhance PCM fusion. The vertical helical shell and tube TES effectively enhances fusion more than the horizontal shell and tube TES. An increase in HTF temperature enhances PCM fusion. Vahidhosseini et al.^42^ analysed the triplex tube TES system using three fin parameters, namely, its shape, arrangement, and count. The bidirectional fins enhanced PCM fusion compared to the unidirectional fins. Kiyak et al.^43^ analysed the effect of curvature and nanoparticles in enhancing PCM fusion. The curvature with 4% Cu nanoparticle reduced the phase change time by 35 and 47% compared to straight fins.

Kenisarin et al.^44^ analysed PCM fusion in a spherical container. Both constrained and unconstrained melting in a spherical container are analysed. The orthogonal fins are found optimal than circumferential fins in enhancing fusion. Hu et al.^45^ analysed a spherical thermal energy storage container with different structured fins, capsule size, and heating temperature. The perforated fins are found with enhanced heat storage due to more PCM. Jia et al.^46^ analysed finned spherical TES using temperature changes, liquid fraction, and cold charging rate. Cold charging duration is reduced by 50% for six fins compared to without fins. Meghari et al.^47^ analysed melting using n-octadecane and gallium in a spherical container. Fins, namely, normal and hollow fins, are analysed to enhance the fusion process. Hollow fins reduce melt time by 14 times compared to those without fins. Fan et al.^48^ analysed PCM melting in a finned spherical container. TES is enhanced when the fin height is increased. Melt duration is reduced by 30% for the highest fin. Conduction and convection near the fin enhance PCM melting.

From the studies, PCM fusion is enhanced using nanoparticles, fins, and various parameters such as fin location, HTF temperature, and concentration of nanoparticles. Some studies are done on a spherical container with fins and nanoparticles. Fins enhance heat transfer for more PCM fusion. Though studies are done on enhancing fusion in a finned spherical container, a comprehensive study about its location in enhancing fusion is less common. The present study is novel since it enhances fusion using a spherical container with fins located near the circumference. The present study identifies the problem behind the melting alleviation. It places the heating area at an optimal location to enhance heat transfer and expedite melting. Fins are placed inside and outside the spherical container to enhance heating by penetration of the heating surface. Four different cases, namely, case 1 with a unfinned spherical container, case 2 with two fins located near the top, case 3 with two fins near the bottom, and case 4 with two fins located near the top and two fins located near the bottom, are analysed. The fusion duration is meagre for case 4, and it fits for application in solar flat plate collectors. Fins’ presence near the top and bottom enhances heat transport, expedites fusion and enhances storage/release rate. The overall fusion time is reduced due to the fins’ location. The present study has several topics, namely, an introduction which discusses the present study on TES, materials and methods, which discuss the materials used and methods followed for carrying out the experiment. It is followed by results and discussion, which discuss the HTF temperature effect, variation in PCM temperature during fusion, storage/release rate, efficiency, and effectiveness. A comparison is carried out at the end. Finally, a conclusion is made on the analysis.

## Materials and methods

### Experimental setup

**Fig. 1:**
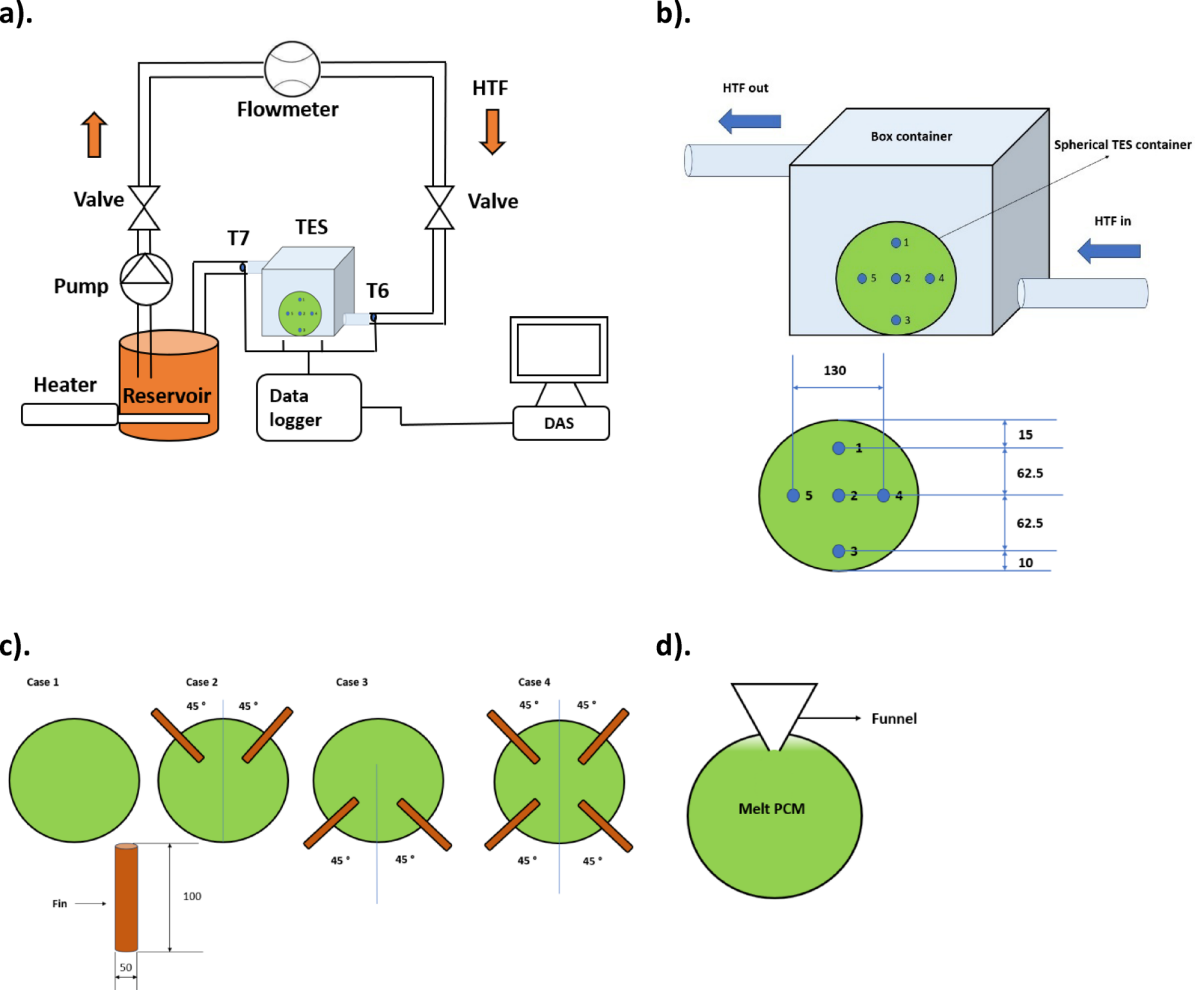
Indicates (**a**). TES setup, (**b**). cuboidal container and thermocouple locations, (**c**). finned spherical TES container, and (**d**). PCM filling in the container.

Fusion analysis is carried out in a spherical TES for applications in solar systems. Figure 1 indicates the experimental schematic with thermocouple location and different cases of spherical TES containers used for the study. The setup consists of valves placed near the entry of the TES system and exit of the reservoir, a flowmeter, a pump, a heater, thermocouples (T1, T2, T3, T4, T5, T6, and T7), a data logger, and a data acquisition system. The reservoir is filled with water and fitted with a heater. The pump is connected to the reservoir through pipes. The exit from the pump is connected to the valve, which controls the flow of water. HTF at the desired flow rate is allowed to flow through the TES using valves and flowmeters. After the flowmeter, a valve is attached to control the flow appropriately. At the end of the valve, the TES system is coupled with the piping system. The TES system houses spherical containers with PCM for energy storage/release. Thermocouples are placed inside (T1, T2, T3, T4, and T5) and at entry and exit locations (T6 and T7) of the TES container.

The TES system has four different cases, namely, case 1—without fins, case 2—two fins near the top with an angular displacement of 90° between each other, case 3—two fins placed near the bottom with a displacement of 90°, and case 4—fins attached both near the top and bottom. The analysis is carried out for all the cases to enhance PCM fusion. The TES system is finally connected to the reservoir. The setup is connected entirely through a piping system made of steel, which is covered by glass wool insulation (0.0343 W. m^−1^. K^−1^). The TES (box container) is covered with insulation, too. The insulation thickness is 30 mm to reduce heat loss. The fin material is chosen as copper to enhance heat transfer. Table 1 indicates the setup specifications.

**Table 1:** TES setup specifications.

Description	Values
PCM	Paraffin wax
Solidus temperature (T_s_)	58 °C
Liquidus temperature (T_l_)	60 °C
Heating coil	5 kW
Pump power rating	0.184 kW
Spherical TES container material	Steel
Heat transfer fluid	Water
Spherical container diameter	150 mm
Box container material	Steel
Spherical container thickness	2 mm
Tube material	Steel
Box thickness	3 mm
Tube diameter	20 mm
Tube thickness	2 mm
Thermal conductivity of insulation	0.0343 W. m^−1^. K^−1^
Insulation	Glass wool
Insulation thickness	30 mm
Fin material	Copper

Figure 1d indicates PCM filling inside the container. PCM filling in the container is done using a funnel. Initially, PCM is melted, and the melted PCM is poured into the container using a funnel. The PCM of mass 1 kg is measured and filled inside the container. Once it is filled, the thermocouples are inserted inside the container and placed at predetermined locations. Then the container is sealed to avoid leakage. The HTF flows from the reservoir connected with the heater. It flows through valves and a pump to reach the TES container at the required conditions. The HTF exit from the TES is connected to the reservoir. The cycle is repeated till complete melting.

### Experimental procedure

The HTF is heated using a submersible heater in a reservoir. The reservoir could hold 25 L of water. HTF is maintained at 70 °C and 75 °C, using a thermostat. Hot HTF is pumped through the valve using a centrifugal pump. HTF flow rate is maintained at 30 LPH using a flowmeter and valve. HTF at the required condition pass through the TES with a spherical container. High-temperature HTF exchanges heat with low-temperature PCM in a spherical container, leading to melting. Thermocouples are located inside the TES container at the centre, top (15 mm below the upper surface), and bottom (10 mm above the bottom surface) to measure temperature changes. It is located at the centre and near the circumference of spherical TES and at the entry and exit of box TES and wired to a data logger and DAS for measuring temperature changes at different periods. The study is carried out for four TES cases, namely, cases 1, 2, 3, and 4. HTF loses some heat with a spherical TES container and flows through a piping system to reach the reservoir. Once melting is done, HTF at 30 LPH and 28 °C flows through TES to solidify PCM. Temperature variations during solidification are measured using thermocouples for fusion analysis.

### PCM

Paraffin wax is opted as PCM for the study. It is chosen based on the availability and application in solar flat plate collectors. Wax, with a melting point of 60 °C, is chosen since it is suitable for applications in solar systems. PCM has high latent heat and low volumetric expansion, hence it is compatible with the system. Though it suffers from meagre thermal conductivity, it possesses high fusion heat required for energy storage applications. Further heat storage/release occurs at near isothermal conditions. Table 2 indicates the thermophysical properties of the wax used for the study.

**Table 2:** Paraffin wax, RT60, thermophysical properties (www.rubitherm.eu)^49,50^.

Property	Value
Thermal conductivity (W. m^−1^. K^−1^)	0.2
Melting temperature (°C)	60
Enthalpy during phase change (kJ. kg^−1^)	190
Specific heat (kJ. kg^−1^. K^−1^)	2.1 (solid) 2.15 (liquid)
Density (kg. m^−3^)	880 (solid) 770 (liquid)
Expansion	12.5%
Flash point (°C)	210
Maximum temperature (°C)	80

### TES container

The study uses a spherical TES container to enhance PCM fusion. Four different cases are used for the study, namely, cases 1, 2, 3 and 4. Thermocouples are placed at the centre, near the top, bottom and side locations to analyse the fusion process. The containers are made of steel with a diameter of 150 mm and a thickness of 2 mm. Thermocouples are inserted through holes in steel balls and then sealed to avoid leakage.

### Performance

The TES container performance is analysed using heat supplied, heat stored/released, storage/release rate, effectiveness, efficiency, and Stefan number.

The heat supplied to TES is^51^,

1 $${\mathrm{Q}}_{{{\mathrm{in}}}} = \dot{m} C_{pw} \left( {T_{wi} - T_{wo} } \right)*t$$

where C_pw_, specific heat (kJ. kg^−1^. K^−1^), $\dot{m}$ is the flow rate (kg. sec^−1^), T_wo_, exit temperature (K), T_wi_, temperature in (K), of HTF, and t, is the time taken (sec).

Energy stored is^52^,

2 $${\mathrm{Q}}_{{{\mathrm{st}}}} = \left\{ {\begin{array}{*{20}c} {m C_{ps} \left( {T_{pcm} - T_{ini} } \right) T_{pcm} < T_{s} } \\ {m \left( {C_{ps} \left( {T_{pcm} - T_{ini} } \right) + F_{i} L T_{s} } \right) < T_{pcm} < T_{l} } \\ {m \left( {C_{ps} \left( {T_{s} - T_{ini} } \right) + F_{i} L + C_{pl} \left( {T_{pcm} - T_{l} } \right)} \right) T_{pcm} > T_{l} } \\ \end{array} } \right.$$

where C_pl_, and C_ps_, liquid and solid PCM specific heat (kJ. kg^−1^. K^−1^), m, PCM mass (kg), T_pcm_, T_ini,_ T_l,_ T_s_, is temperature (K), initial temperature (K), liquidus and solidus temperature (K), L, latent heat (kJ. kg^−1^), and F_i_, melt fraction of PCM, respectively. As melting occurs, the average temperature increases. If the temperature is below the melting temperature, the actual mass m is considered along with the heat capacity of the solid PCM. If the average temperature falls in the range of 58 to 60 °C, it is considered a mushy zone. Hence, melt fraction and latent heat are considered with actual mass. After melting, the PCM mass, along with the specific heat capacity of liquid PCM with temperature rise, is considered. Hence, actual mass remains the same, but the parameters like average temperature will change along with heat capacity and latent heat during heat storage calculation. Though the mass of liquid PCM is different from that of solid PCM, the overall PCM mass remains the same. The parameters are added according to the average temperature rise during melting for heat storage calculations. The melt fraction is^52^,

3 $$F_{i} = \frac{{T_{pcm} - T_{s} }}{{T_{l} - T_{s} }}$$

The efficiency is^53^,

4 $$\eta = \frac{{Q_{st} }}{{Q_{in} }}$$

The effectiveness is^54^,

5 $$\epsilon = \frac{{Q_{st} }}{{Q_{max} }}$$

$Q_{max}$ is the maximum heat stored. It is attained after complete melting. The heat stored will be maximum after complete PCM melting.

Stefan number is^55^,

6 $${\mathrm{Ste}} = \frac{{C_{p} \left( {T_{PCM} - T_{ini} } \right)}}{L}$$

### Uncertainty analysis

The uncertainty in measurements during PCM fusion is analysed. The components opted for the study are with accuracy constraints, hence an uncertainty analysis is made. Table 3. Indicate the components’ uncertainty used for the study. The uncertainty is carried out using^56,57^. The uncertainty is given by,

7 $$\Delta Y = \sqrt {\sum \left( {\frac{\delta Y}{{\delta X_{i} }} \Delta X_{i} } \right)^{2} }$$

**Table 3:** Uncertainty with instruments and their ranges.

Parameter	Instrument	Uncertainty	Range
Flowrate (LPH)	Flow meter	± 1.5 LPH	0–200
Mass (g)	Mass balance	± 0.01 g	0–600
Temperature (ºC)	K-type thermocouple	± 0.5 °C	− 200–1000
Heat stored (kJ)		± 2 kJ	
Efficiency (%)		± 1%	
Effectiveness		± 0.012	
Ste		± 0.0055	

where *δY* is the derived uncertainty, *ΔY* is the overall uncertainty, and *δX* is the measured uncertainty. Table 3 indicates the measurement uncertainties. The overall uncertainty is around 3% agreeable with the existing literature^58^.

## Results and discussions

This section discusses about PCM fusion process in a finned spherical container, the temperature variation during fusion, heat stored/released, storage/release rate, efficiency and effectiveness. A detailed analysis is carried out to study fusion enhancement in a finned spherical container, along with a comparative analysis.

### Fusion in a finned spherical container

PCM fusion is analysed in a spherical container to identify the optimal case for enhancing the heat storage/release process. The spherical TES container with fin incorporation is used for the study. Four different cases, namely, cases 1, 2, 3, and 4, are used for the analysis. Case 1 is analysed at the start, and it is further extended to other cases. Fusion is studied using HTF at 70 °C, 75 °C, and 28 °C at a 30 LPH flow rate. Hot HTF at 70 °C, 30 LPH enter TES in a box container. Heat exchange takes place due to the difference between the PCM and HTF temperatures. PCM starts melting near the container circumference due to heat exchange. It melts aggressively near the circumference due to more heat transfer. Hence, more PCM melts in a short duration, and it starts accumulating near the container’s top.

Solid PCM with higher density melts leading to the formation of liquid PCM with lower density. This density variation leads to liquid PCM accumulation near the top and solid PCM near the bottom. Solid and liquid PCM movement to the bottom and top creates natural convection for effective melting. It indicates natural convection is dominant during melting^36^. During the movement, molten PCM exchanges heat with solid PCM, and melting becomes concomitant. Since heat transfer takes place throughout the circumference, melting takes place near the container surface initially. As more PCM melts, it pushes solid PCM to the bottom and centre. Hence, solid PCM accumulates near the bottom centre. Melting in a container occurs from the circumference to the centre. A feeble conduction is found near the bottom centre due to solid PCM.

PCMs’ meagre thermal conductivity contributes to this effect. Though solid PCM segregate near the centre, it is short-lived since effective heat transfer occurs throughout the circumference. The superheated molten PCM transports heat to the solid PCM, resulting in aggressive melting and expansion. PCM solidification is done after melting. PCM after melting is at a high temperature, leading to more heat transport to HTF at a low temperature. HTF at low temperature absorbs more heat from the superheated liquid PCM through the spherical container surface. Aggressive heat transfer takes place due to a large temperature difference, leading to solid PCM formation near the container circumference. Since solid PCM has less thermal conductivity, heat transfer is resisted through PCM. Hence, more liquid PCM traps inside the container in the inner space. However, highly dense solid PCM settle down, leaving liquid PCM near the top, leading to solidification expedition. PCM solidification is dominated by conduction, takes more time for solidification due to its meagre thermal conductivity. PCM solidification expedites in a spherical container due to heat transfer from the container surface.

PCM fusion analysis is done for case 1 with an unfinned spherical container initially. PCM melting is more near container circumference due to heat exchange between the PCM and hot HTF. More melting occurs due to molten PCM movement resulting in natural convection for effective melting. Melting is alleviated due to fin absence. During solidification, solid PCM forms near the container’s inner surface, and further solidification is resisted. However, dense solid PCM settles, and liquid PCM occupies near the top, leading to solidification expedited due to more heat exchange. The absence of an inner heat transfer area reduces solidification in case 1. But this is addressed in other cases.

PCM melting is analysed for case 2 with fins near the top. This is effective since heat penetrates deep into the container’s inner space. HTF at high temperature exchanges heat with copper fins and a steel container. PCM melts near the circumference initially. Further, due to the presence of fins that penetrate deep inside the steel container, melting is expedited. Fins with high temperature transfer heat through conduction. This heat penetrates deep inside the container and exchanges heat with the PCM, leading to melting. PCM melting is expedited in the upper region due to the fins. It leads to reduced melt time. However, PCM settles, which takes much time to establish complete solidification.

The fins’ deep penetration inside the container forms more liquid PCM near the top and more melting. During solidification, solid PCM settling and liquid PCM movement to the top expedites solidification in case 2 due to fins. Liquid PCM is solidified due to the fins’ presence. Fins exchange heat with PCM, leading to more solidification. Fins’ presence in the upper region is effective during solidification since liquid PCM solidification is expedited. Fusion is studied for case 3 with the upper region devoid of fins. Melting initially occurs near the container circumference, pushing solid PCM to the bottom. Settled solid PCM stays for less time since heat transfer areas like fins are positioned near the bottom. Fins absorb heat from HTF, and they conduct heat inside the spherical TES container housing PCM. Fins are extended in the inner and outer regions of the container. Hence, heat is transferred from HTF in the outer region to PCM in the inner region. It leads to a melting expedition since settling is reduced due to fins. Hence, case 3 has enhanced melting compared to cases 1 and 2 due to optimal fin location near the bottom^28^. PCM solidification is done after melting.

Initially, PCM solidifies near the container circumference and settles. The presence of fins near the bottom is less optimal since more liquid PCM to be solidified segregates near the top. Heat transfer area absence near the top enhances solidification time. Hence, case 2 has a solidification expedition than cases 1 and 3. Case 4 is studied to enhance PCM fusion. Case 4 is with fins near the top and bottom, enhancing heat transfer. During melting, PCM melts near the circumference, and solid PCM settles. This is alleviated using fins near the bottom. Fins near the bottom enhance melting. Heat penetrates inside the PCM placed in a spherical container through fins. Fins transfer heat through conduction, leading to more melting.

The fins’ presence near the top further enhances PCM melting at initial stages, and overall melting is expedited in case 4 more than in the others. Solidification occurs from bottom to top; hence, a heating area near the bottom enhances solidification at the initial stage. Further solidification is enhanced due to the top fins. Solidification is alleviated as molten PCM accumulates near the top. However, case 4 has fins near the top, enhancing solidification due to more heat transport. Hence, case 4 has less overall fusion time than the other cases due to optimal fin location.

**Fig. 2:**
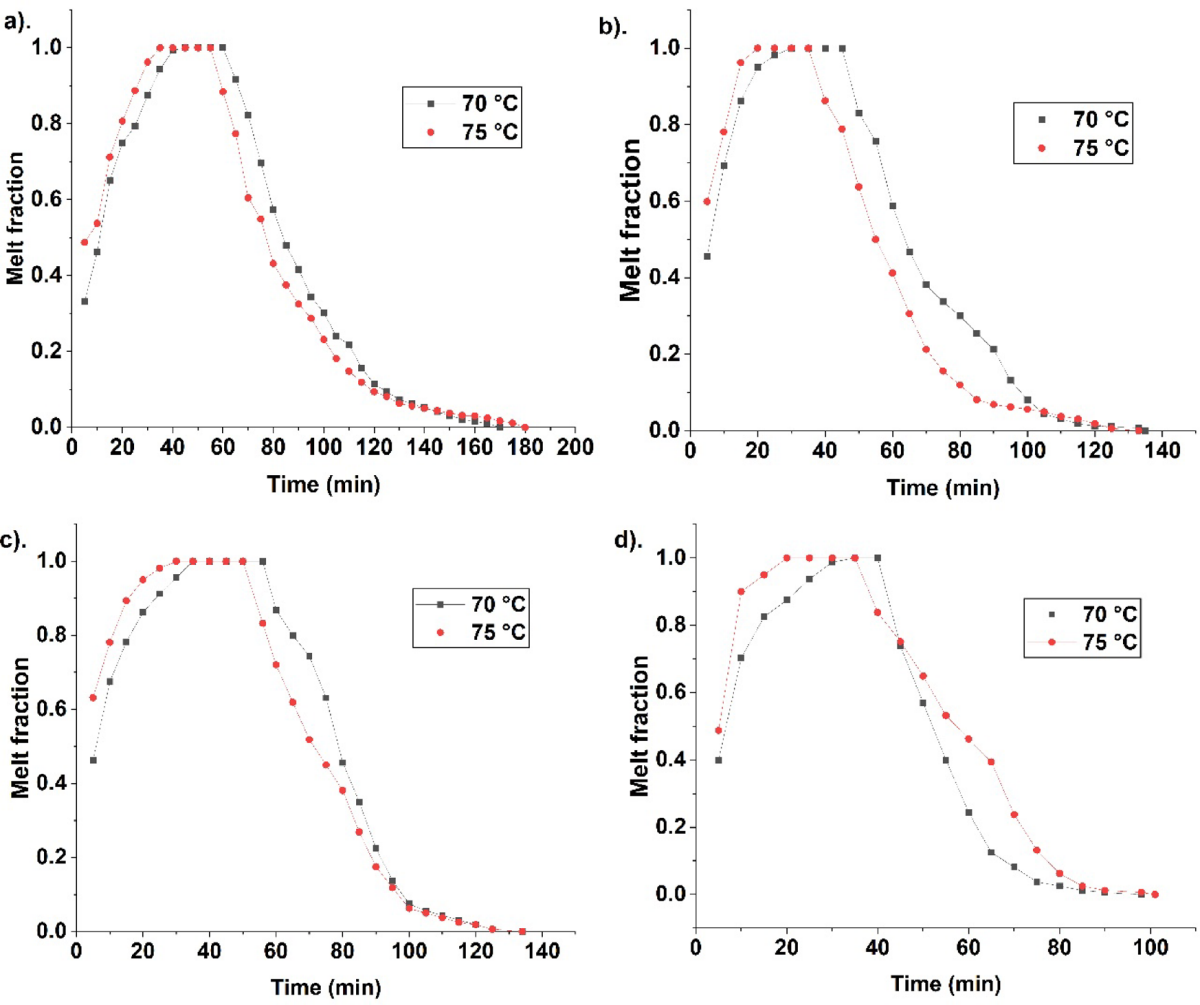
Indicate melt fraction variation during PCM fusion for different cases namely, (**a**) case 1—Unfinned, (**b**) case—2 top fins, (**c**) case 3—bottom fins, and (**d**) case—4 top and bottom fins, at HTF temperatures 70, and 75 °C.

Figure 2 indicates the melt fraction at various periods. It indicates the melt fraction expedition for case 4, followed by case 3, case 2, and case 1 during melting. The optimal fin’s location in case 4 effectively transfers heat and enhances melting. Four fins in case 4 are at the optimal location, hence enhancing PCM melting. It reduces settling and increases overall fusion. The curve for case 4 is steep, indicating effective melting. The curves for cases 3, 2, and 1 are less steep than case 4, indicating melting expedition in case 4. The fin location near the top and bottom enhances PCM melting due to the heating area. Fins enhance heat transfer due to conduction and heating area penetration inside the container. The penetration leads to heat transfer in deep areas of solid PCM accumulation. It leads to more melting in a short time since the heating area is available at an optimal location. The melt PCM results in further solid PCM melting due to heat exchange between the fin and superheated molten PCM. Hence, the curve is steeper for case 4 than the others. Initial heat transfer occurs through conduction in the PCM, and it then changes to convection due to the movement of the molten PCM^59^. During solidification, case 4 is more steeply downward, indicating more fusion expedition. The steepness is more for case 4 than for cases 3, 2, and 1. The presence of fins at optimal locations near the top and bottom results in heat transport. It increases overall fusion. The increase in natural convection, coupled with conductive heat transfer from fins, enhances overall fusion in the spherical container. The melt fraction after 10 min of melting is 0.463, 0.675, 0.693, and 0.703, for cases 1, 2, 3, and 4, at 70 °C HTF temperature. High melt fraction is seen in case 4 due to enhanced melting compared to the others. The optimal fin location for case 4 at the top and bottom results in aggressive melting. Hence, the melt fraction is higher in case 4 than others. Though the heating area is present in cases 2 and 3, the location is optimal in case 4. Case 1 is with no fins, resulting in less melt fraction due to less heat surface and natural convection. Though heat transfer occurs throughout the circumference, melting is low in this case. Absence of fins results in melting alleviation. Further, cases 2 and 3 possess a heat surface near the top and bottom. But the fusion rate is less in these cases since the heat surface is non-optimal during fusion.

A higher melt fraction is seen in case 4 than other cases. It indicates effective fusion in case 4. At 75 °C HTF temperature, the melt fraction is 0.537, 0.781, 0.781, and 0.9, for cases 1, 2, 3, and 4. A high melt fraction is seen in case 4, indicating melting expedition. The location of fins enables effective heat transfer in case 4, leading to a higher melt fraction in a short time. Whereas other cases possess non-optimal fin location, leading to less melt fraction. Further fins’ presence enhances conductive heat transfer and results in more natural convection for effective PCM melting. As conduction is enhanced due to fins, it results in melting. As PCM melts, the liquid PCM move to the top due to density variation, resulting in natural convection. Though fins obstruct PCM movement, the overall melting enhances due to its penetration. 75 °C is found with high values due to the temperature difference and melting. During solidification, the melt fraction after 10 min of solidification at 70 °C is 0.822, 0.8, 0.756, and 0.568, for cases 1, 2, 3, and 4. Case 4 has a lower melt fraction due to effective heat transfer, favourable fins location, and more heat transfer throughout the circumference. For 75 °C, the melt fraction is 0.773, 0.7206, 0.788, and 0.751 for cases 1, 2, 3, and 4, indicating case 4 is effective in enhancing fusion. PCM solidification requires a heat surface at an optimal location. Case 4 has high solidification both at 70 °C and 75 °C due to optimal fin location. During solidification, solid PCM settle; hence, a heating area near the top enhances solidification. Fin location near the top and bottom ensures solidification expedition in case 4. Whereas in case 1, solidification takes time to establish due to the absence of fins and liquid PCM accumulation near the top. Case 2 is with fins near the top. Hence, solidification is expedited. In case 3, fins are restricted to the bottom, which is non-optimal since liquid PCM to be solidified accumulates near the top. Further, the melt fraction is less at 75 °C due to enhanced heat transport. The high temperature HTF leads to more melting and a temperature rise in 75 °C, leading to aggressive solidification as a result of the temperature difference between HTF and PCM. Case 4 is with more melting due to conductive and convective heat transfer. Fins enhance conductive heat transfer, while the PCM movement during melting increases convective heat transfer. During solidification, the conductive heat transfer due to fins enhances resulting in fusion expedition.

### Temperature distribution during fusion

**Fig. 3:**
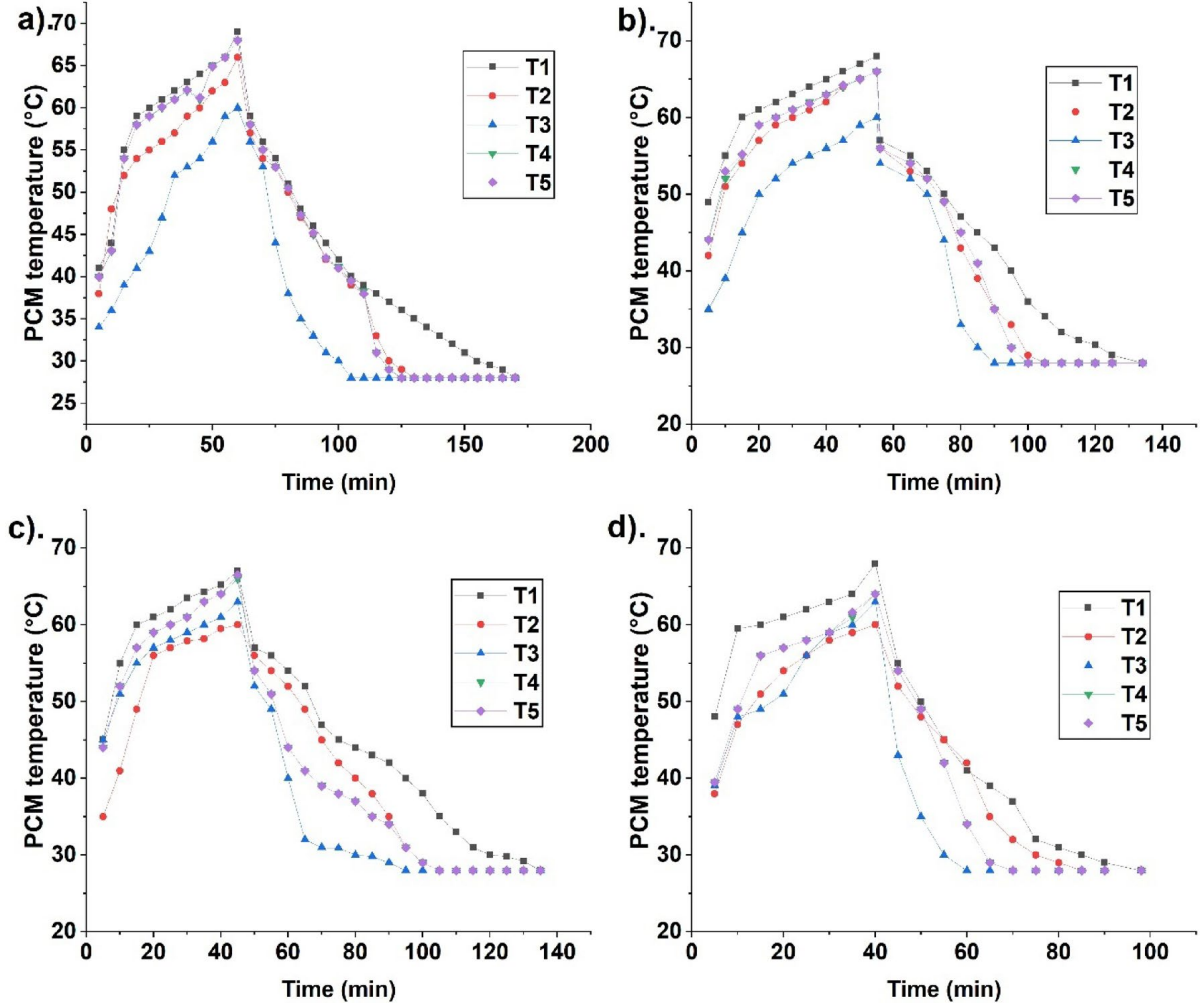
PCM temperature variation for 70 °C HTF temperature for different cases, namely, (**a**) case 1—unfinned, (**b**) case 2—top fins, (**c**) case 3—bottom fins, and (**d**) case 4—top and bottom fins.

PCM temperature changes due to heat distribution. Figure 3 indicates the PCM temperature distribution for different cases at 70 °C HTF temperature. PCM melting is high due to a greater temperature difference at the initial stages between HTF and PCM in a spherical container. As more PCM melts, it accumulates near the top, leading to a higher temperature at the top than at the bottom. This movement of PCM creates natural convection. The PCM Temperature distribution is indicated using thermocouples placed at different locations in a container. Thermocouples placed near the top indicate a higher temperature than at the bottom due to more superheated liquid PCM near the top.

The temperature near the top reaches melting early due to the liquid PCM. It increases further and gets closer to the HTF temperature. The temperature at the centre reaches the melting point after the top due to the solid PCM for melting. Finally bottom thermocouple reaches the melting temperature, indicating complete PCM melting. Thermocouples placed radially near the circumference attain the melting point before the centre due to liquid PCM formation. During solidification, the temperature near the bottom attains solidification early.

It indicates solidification occurring from bottom to top. As more solid PCM forms, the temperature near the centre attains solidification. Finally, the top reaches solidification. Thermocouples placed radially near the circumference attain solidification after the centre thermocouples due to less PCM solidification. Temperature distribution is analysed for four cases (cases 1–4) during PCM fusion.

**Fig. 4:**
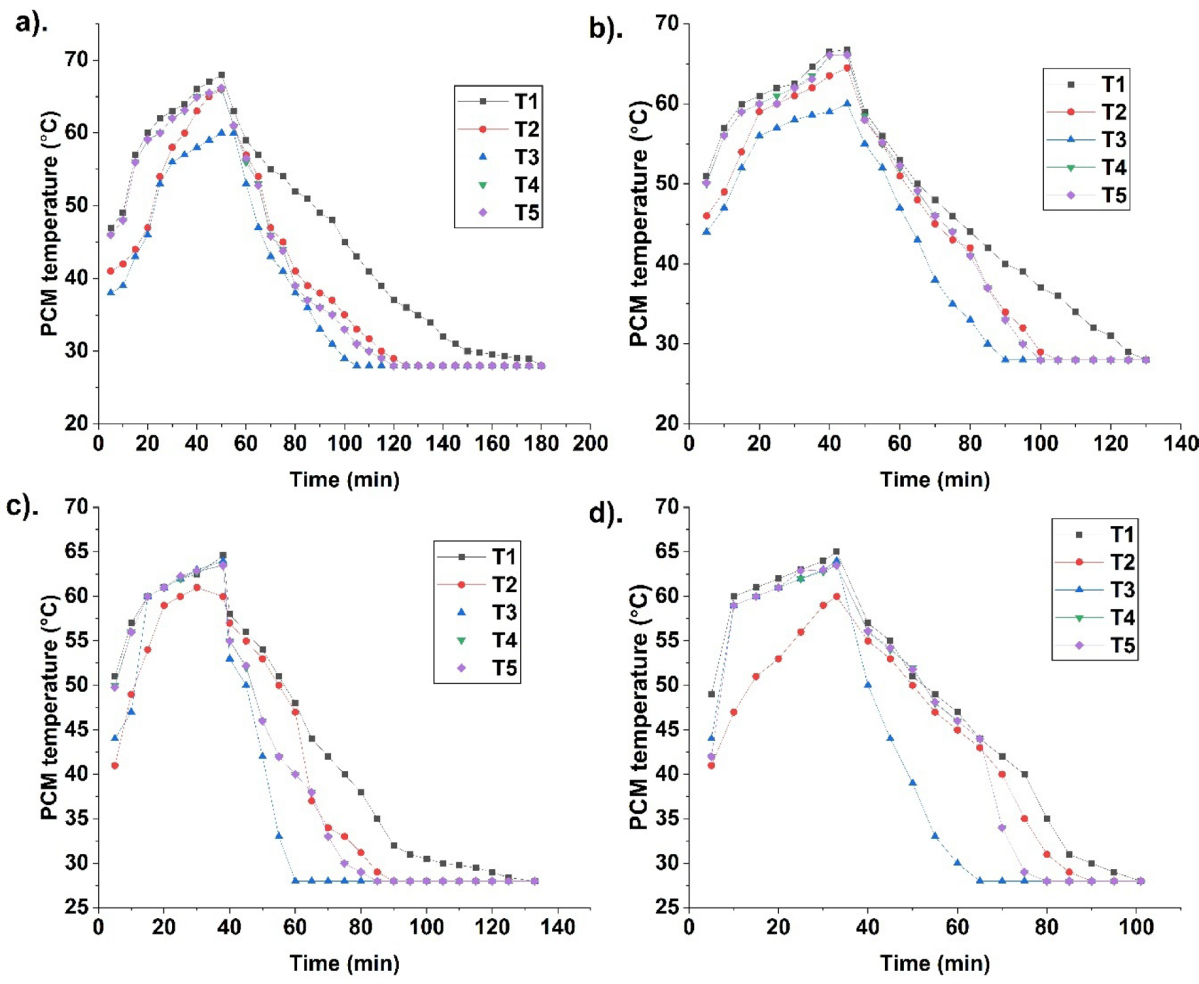
PCM temperature variation for 75 °C HTF temperature for different cases, namely, (**a**) case 1—unfinned, (**b**) case 2—top fins, (**c**) case 3—bottom fins, and (**d**) case 4—top and bottom fins.

**Fig. 5:**
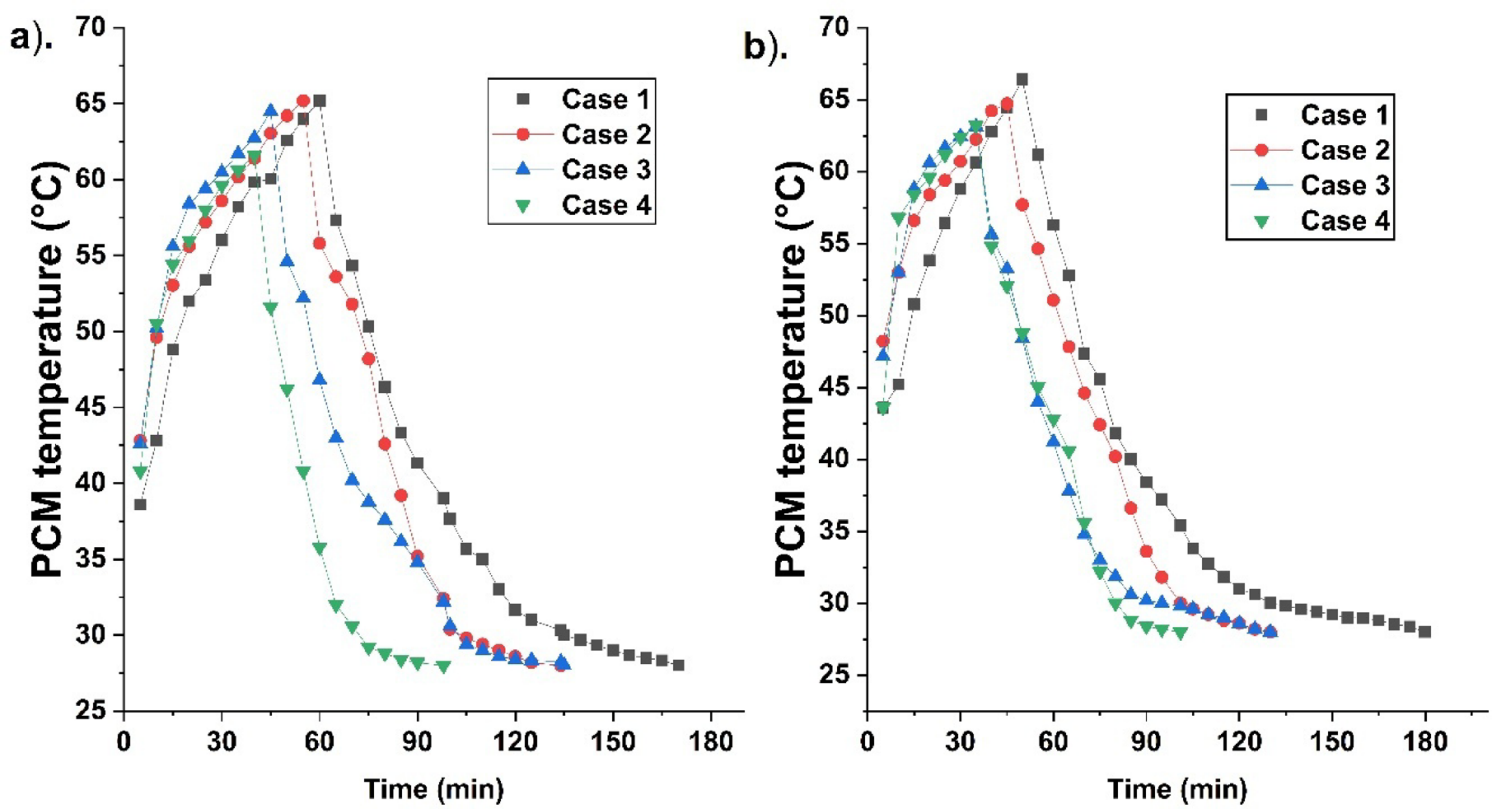
Indicate the average PCM temperature for different cases (1, 2, 3, and 4), (**a**). at 70 °C and (**b**). 75 °C HTF temperature.

Figure 4 indicates PCM temperature for different cases 1, 2, 3, and 4, at 75 °C. Case 1 is a finless configuration. This case takes more time to melt due to a smaller heat transfer area than others. Here, the temperature is found to be high near the top and low near the bottom. Low temperature indicates less melting. As more PCM melts temperature near the top increases rapidly and attains the HTF temperature. The temperature near the radial location attains melting after the top since the PCM melts aggressively. The temperature near the bottom reaches melting at the end. It indicates complete melting. The temperature attains solidification earlier near the bottom. It attains solidification later in the centre, radial, and top. The top region attains solidification at the end, indicating liquid PCM is closer to the top. Figure 5 indicates the average temperature for different cases at 70 °C and 75 °C HTF temperatures.

Case 2 temperature distribution is analysed during PCM fusion. Case 2, with two fins placed near the container’s top, enhances PCM melting. Melting is aggressive at the start due to the fins’ presence, leading to more liquid PCM and temperature near the top. High temperature is reached more swiftly than in case 1 due to the fins’ presence. The temperature rises steadily till it reaches close to the HTF temperature. The temperature distribution is high near the top and decreases toward the bottom. The bottom region has feeble conduction due to more solid PCM, leading to less temperature rise. Absence of fins further reduces melting, and hence the overall melting duration is more but less than that of case 1. The temperature at the radial location reaches the melting point before the centre due to more melting. During solidification temperature is meagre near the bottom due to solid PCM. It is more near top due to more liquid PCM. Complete solidification is ensured when the temperature near the top reaches solidification.

Case 3 has two fins near the bottom. Fin presence near the bottom enhances melting, leading to less PCM accumulation. PCM melts near the bottom and circumference, and moves to the top, thereby raising the temperature near the top. PCM settling is reduced, resulting in more temperature rise. The container centre reaches a high temperature at the end, indicating solid PCM segregation. The temperature at the radial location is high due to more liquid PCM. Temperature is more near circumference, and it is low at the centre, indicating melting occurs radially. High temperature is found near the top due to more superheated liquid PCM. During solidification, PCM solidifies near the circumference, and it grows toward the centre. More solidified PCM sinks due to high density and gravity. Hence, more solidified PCM is found near the bottom at a lower temperature. Solid and liquid PCM movement leads to more heat exchange for effective solidification and temperature reduction. Further, in case 3, fins are located near the bottom, which increases solidification time since liquid PCM accumulates near the top. The fin’s absence reduces heat transfer and solidification. Hence, solidification takes longer than melting. Case 3 enhances melting due to the fins’ location; however, solidification is alleviated compared with case 2.

Case 4 is analysed for temperature distribution. Fins’ presence near the top and bottom enhances melting. Hence, temperature increases rapidly. However, the temperature is high at the top due to liquid PCM. The temperature near the top and bottom reaches melting early, with the centre reaching it at the end due to solid PCM. The temperature near radial locations reaches melting early due to molten PCM. The temperature at all locations reaches melting early in case 4 due to enhanced melting. The fins’ location is optimal for enhancing melting. Solidification temperature variation is studied after melting. Temperature at locations like the top and bottom reaches solidification early due to the presence of fins. However, the temperature at the centre reaches solidification at the end due to ineffective heat transfer. The fins’ presence enhances heat transfer near the top and bottom, leading to solidification and less temperature. Hence, case 4 is with a fusion expedition rather than the other cases.

The average temperature is high for case 1 during melting due to sensible heat. Enhanced melt time leads to increased heat addition and temperature. The average temperature is lower for case 2 than for case 1 due to the fins’ presence. The melting expedition in case 2 results in less sensible heat and temperature. Case 3’s average temperature is lower compared to case 2 since the fins’ location is optimal in enhancing fusion. Case 4’s average temperature is less than other cases due to the melting expedition.

Figures 3 and 4 indicate the temperature distribution in all cases (cases 1, 2, 3, and 4) at different locations in the spherical TES container. It indicates a higher temperature rise near the top (T1) than at other locations due to liquid PCM. Hence temperature trend is steep. It is followed by T4, T5, and T2, which are located near the circumference and at the centre. Since liquid PCM has a lower density, it moves to the top, hence a higher temperature is indicated at this location. This movement of solid and liquid PCM creates natural convection for effective melting. The temperature gets close to the HTF temperature at these locations due to liquid PCM and superheating. The temperature trend is steeper for 75 °C due to the temperature difference and melting. During solidification curve trend is steeply downward at the bottom locations. The solidified PCM has a lower temperature, hence the temperature T3 reaches solidification in a short time. The temperature T2 reaches solidification after T3. T1 reaches solidification at last, indicating complete solidification. The trend is more steeply downward for T3 due to effective solidification at this location. From Fig. 3, the temperature after 10 min melting for case 1 at T1, T2, T3, T4, and T5 are 44, 41, 36, 43, and 43.1 °C. High temperature is displayed at location T1 due to more melting. Since melting occurs from the top, high molten PCM is seen in location 1 at the top. It is followed by T2 and T3. The T4 and T5 are higher than T2 and T3 since they are located near containers centre. For cases 2, 3, and 4, the temperatures at T1, T2, T3, T4, and T5 during melting are: 55, 51, 39, 52, and 53 °C; 55, 41, 51, 52, and 52 °C; 59.5, 47, 48, 49 and 49 °C, respectively. The results indicate a high temperature for case 4 at location 1. The lower temperature at the other location for case 4 is due to the melting onset. There is a vigorous melting seen after this time.

The fin location near the top and bottom in case 4 enhances PCM melting due to the heating area at the optimal location. Further, the conductive and convective heat transfer enhancement results in effective PCM melting. During solidification after 10 min, the temperature at T1, T2, T3, T4, and T5 for case 1 is: 56, 54, 53, 55, and 55 °C. Low temperature is seen at location 3 at the bottom. This indicates solid PCM settling near the bottom. Hence, the bottom temperature is low. Further, the middle locations are at high temperature since fusion is occurring. Temperature at T1, T2, T3, T4, and T5 for cases 2, 3, 4, and 5 are: 55, 53, 52, 54, and 54 °C; 56, 54, 49, 51, and 51 °C; 50, 48, 35, 49, and 49 °C, respectively. Location T1 is with high temperature due to more melted PCM. Further, other locations are at low temperature since PCM start solidifying and settles near the bottom.

From Fig. 4, the temperature at locations T1, T2, T3, T4, and T5 for 75 °C during melting are 49, 42, 39, 48, and 48 °C. High temperature is seen in location 1 due to more PCM melting and accumulation of molten PCM. T3 is with low temperature due to PCM settling. T4 and T5, near the container circumference and in the middle, are found with more temperature than T3 due to more molten PCM at the location. For cases 2, 3, and 4, the temperatures are: 57, 49, 47, 56, and 56; 57.5, 49.7, 47.4, 56.5, and 58.5; 58, 48, 59, 59, and 59; respectively. The results reveal that case 4 has with high temperature than cases 1, 2 and 3 due to optimal fin location enhancing heat transfer. T1 is at a higher temperature than the others due to more melting near the top. Fin enhances conductive heat transfer while PCM melting is influenced by convection. It results in aggressive melting in case 4 due to the presence of fins both in the top and bottom. The curves for case 4 were steeper, indicating effective heat transfer and melting. The curves for all other cases are less steep, indicating less favourable fin locations for heat transfer.

Figure 5 indicates the average temperature variation at different periods for cases 1, 2, 3, and 4. Case 1 is found with a higher temperature after melting than the others due to the alleviated melting than others. It leads to less melting and more sensible heat addition. As a result, it increases the PCM temperature more than others. The temperature increase is more in 70 °C than in 75 °C due to melting alleviation. The temperature after 10 min of melting is 42.82, 49.6, 50.2, and 50.5 °C for cases 1, 2, 3, and 4 at 70 °C. For 75 °C, temperatures are 45.2, 53, 53, and 56.8 °C for cases 1, 2, 3, and 4. High temperature rise is found for case 4 than the others due to melting. The temperature after melting is 65.2, 65.2, 64.5, and 61.6 °C for 70 °C. For 75 °C, it is 66.39, 64.72, 63.12, and 63 °C for cases 1, 2, 3, and 4. It indicates a higher temperature for case 1 due to more sensible heat addition. The prolonged melt time for case 1 leads to more sensible heat addition, hence the temperature after complete melting is high. Further, case 1 is with low natural convection and conductive heat transfer. The curve is steep for case 4, indicating effective melting leading to high temperature. More melting is due to conductive and convective heat transfer. The curves are less steep for cases 1, 2, and 3, indicating less melting compared with case 4. Further cases 1, 2, and 3 are with a heating area at a less favourable location. It leads to more melt time and sensible heat addition. During solidification, the temperature after 10 min at 70 °C is 54.33, 53.6, 52.2, and 46.2 °C, for all cases (1–4). It indicates a lower temperature for case 4 than the others due to effective solidification. For 75 °C, it is 56.3, 54.64, 53.24, and 52.04 °C, for all cases (1–4). Lower temperature for case 4 indicates a fusion expedition. Melting is expedited at 75 °C than at 70 °C due to the temperature rise^35^.

### Heat stored/released

**Fig. 6:**
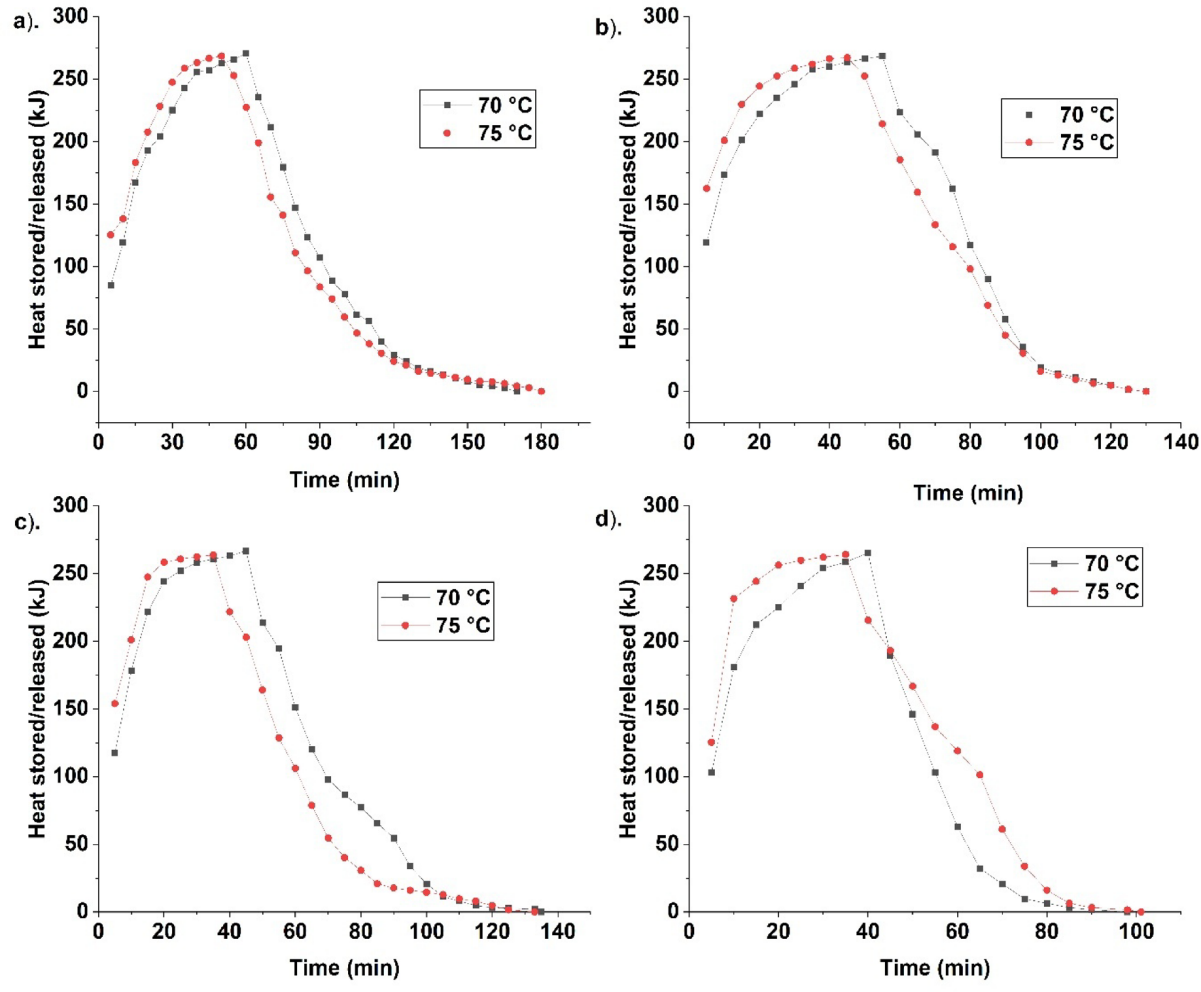
Indicate the heat stored and released for different cases, namely, (**a**) case 1—unfinned, (**b**) case 2—top fins, (**c**) case 3—bottom fins, and (**d**) case 4—top and bottom fins at 70 °C and 75 °C HTF temperature.

PCM fusion leads to heat storage/release. Heat is stored as sensible and latent. Sensible heat is stored before and after melting, while latent heat is stored during phase change. PCM latent heat is high, and it occurs in near-isothermal conditions. Four different cases are studied to analyse heat storage/release. Case 1 is initially analysed. Figure 6 indicates the heat stored/released for different cases at 70 and 75 °C HTF temperatures.

Case 1 is an unfinned container; hence, complete melting takes time to establish. It absorbs more heat for complete melting. Though latent heat remains the same, the sensible heat addition is more. The alleviated melting in a spherical container in case 1 leads to more heat addition in the form of sensible heat. PCM settling, leading to prolonged melting, results in more heat addition. Further, case 1 is with low conductive and convective heat transfer than the others due to no fins and ineffective melting. Heat release is effective in case 1. Since the heat stored after melting is high, the temperature rise is high in case 1. It results in heat transfer. Hence, heat release is effective in case 1 due to prolonged melting and high final temperature as a result of less convective heat transport.

**Fig. 7:**
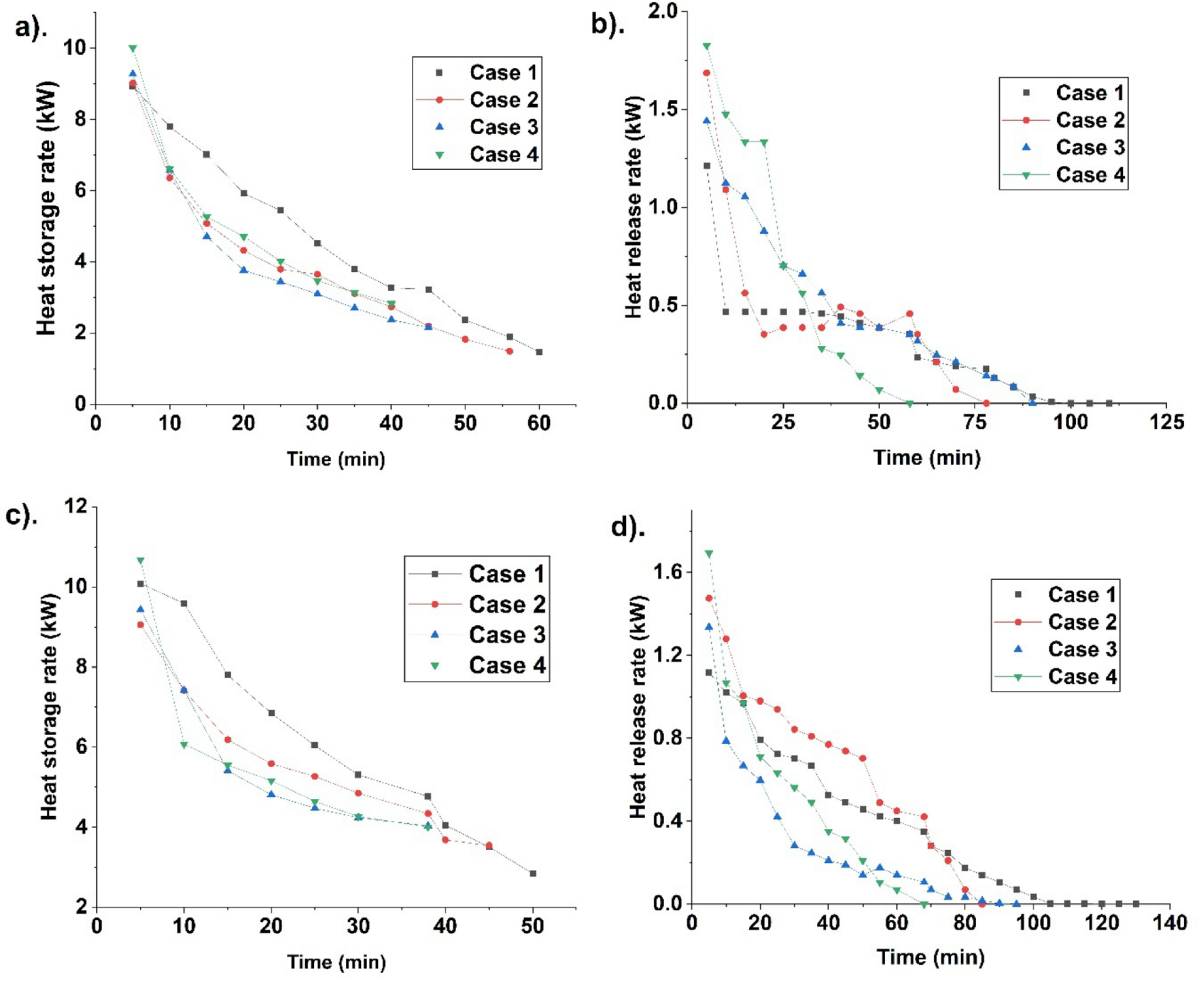
Indicate heat storage and release rate at 70 °C (**a**, **b**) and 75 °C (**c**, **d**) during melting and solidification for different cases.

Case 2 is studied after case 1. The complete melting occurs in a shorter time. Hence, sensible heat is less in case 2. This leads to less temperature rise than in case 1. Further, the presence of fins in case 2 enhances melting, leading to less overall temperature rise. Lower temperature after complete melting leads to less solidification. Hence, heat release takes more time in case 2 than in case 1. Case 3 is further analysed to study its heat storage/release. Case 3 is found with less melting time due to the optimal fin location in enhancing PCM melting. This results in less sensible heat. Temperature after melting is meagre compared to cases 1 and 2. It results in a longer solidification duration due to lower temperatures after complete melting. Though fins are present, it is not optimal in enhancing solidification, leading to their alleviation. Case 4 is analysed for heat storage/release. Case 4 has fins near the top and bottom. Figure 7 indicates the heat storage/release rate for different cases at 70 °C and 75 °C HTF temperatures.

It leads to a fusion expedition due to the fin location. Melting time reduction leads to less sensible heat and lower temperatures after melting. The fins’ location is responsible for this cause. Fins’ location leads to more conductive heat transfer, and it results in enhanced natural convection. This lower temperature after complete melting should have increased the solidification duration. But the fins’ optimal location enhances solidification. Hence, case 4 is optimal in fusion enhancement. The heat storage though less in case 4 when compared with other cases, but its solidification is expedited due to optimal fin location.

From Fig. 6, the curve is steeper for case 4 than for cases 3, 2, and 1. It indicates that effective heat storage is greater in case 4 than others. However, the heat stored is less in case 4 due to less sensible heat. Heat stored for case 1 is higher than the others due to melting alleviation and sensible heat. Heat stored after 10 min of melting is 119, 173, 178, and 180 kJ for cases 1, 2, 3, and 4 at 70 °C HTF temperature. High heat stored is seen in case 4 due to the melting expedition. Fins’ favourable location enhances heat transfer and expedites melting. It leads to more heat storage in less time due to effective natural convection. Hence, case 4 is with high heat storage. At 75 °C, the heat stored is 138, 200, 200, and 231 kJ, for all cases (1–4). High heat storage is seen in case 4 due to the melting expedition. The large temperature difference between PCM and HTF leads to more heat storage in a short time. Further, the fins’ favourable location in case 4 increases heat storage. Heat absorbed after melting is 270, 268, 266, and 265 kJ, for all cases (1–4) at 70 °C. Heat stored is high for case 1 due to melting alleviation and more sensible heat addition. At 75 °C, the heat stored is 268, 267, 263, and 264 kJ for cases 1, 2, 3, and 4. High heat storage is seen in case 1 due to prolonged melting. The heat stored for all cases is high at 70 °C due to more melt time, leading to more heat addition.

The heat release for all cases is analysed. For 70 °C, the heat release after 10 min was 211, 205, 194.5, and 146.2 kJ, respectively, for Cases 1, 2, 3, and 4. The lower value for case 4 indicates effective heat transfer and expedited melting. For 75 °C, the heat release was 227, 214, 202, and 193 kJ. Less value is displayed for case 4, indicating more PCM is solidified. The favourable fins’ location with enhanced overall heat transfer led to solidification expedition in case 4. It is further indicated by a steep trend in case 4.

Heat storage/release rate is analysed for all the cases (1- 4). Case 1 has a lower storage rate due to more melting time. It’s finless, hence heat transfer is less compared with others. It leads to a lower storage rate and more melt time due to less conduction and natural convection. The heat release rate is studied after storage. Though heat release is more due to the temperature difference, solidification takes time to establish. The fins’ absence alleviates the release rate. Case 2 is analysed for the heat storage. The storage rate is higher for case 2 than for case 1 due to the presence of fins. Fins penetrate inside the PCM, leading to effective heat transport. It leads to higher storage. The release rate is high due to fins. It leads to solidification expedition. Further, case 3 is studied for the storage rate. Case 3 has optimal fin location during melting. Hence, the storage rate is higher than in cases 1 and 2, leading to more melting. But during solidification fins’ location is non-optimal since liquid PCM accumulates near the top, devoid of fins. Hence, the release rate is low compared with case 2. Further, case 4 is analysed for the storage rate. It has fins at favourable locations. Hence fusion time is less. The optimal fins’ location leads to a higher melting and storage rate. Heat transfer due to the fin leads to more storage. The release rate is high due to the fins’ location. The fins’ location at the top and bottom leads to more release. Hence, case 4 has a higher heat storage/release rate than the other cases.

From Fig. 7 heat storage/release rate is high at the start due to a temperature difference, leading to heat transport. Heat storage rate at start for different cases (1–4) at 70 °C HTF temperature is 8.92, 9.02, 9.27, and 10.01 kW. For 75 °C, the storage rate is 10.08, 9.05, 9.43, and 10.68 kW for cases 1, 2, 3, and 4. Case 4 has a high storage rate, indicating it is more effective in enhancing heat storage than others. Hence, melting is expedited in case 4. The release rate for different cases 1, 2, 3, and 4, at 70 °C, is 1.21, 1.68, 1.44, and 1.82 kW. For 75 °C, the release rates are 1.11, 1.47, 1.33, and 1.69 kW, for cases 1, 2, 3, and 4. The heat release rate is high for case 4. Both the heat storage/release rate is high for case 4, indicating it is effective in enhancing fusion. The high storage/release rate for case 4 is due to more conduction because of the optimal fins location. The conductive heat transport results in more melting, and it becomes concomitant due to natural convection.

### Performance analysis

**Fig. 8:**
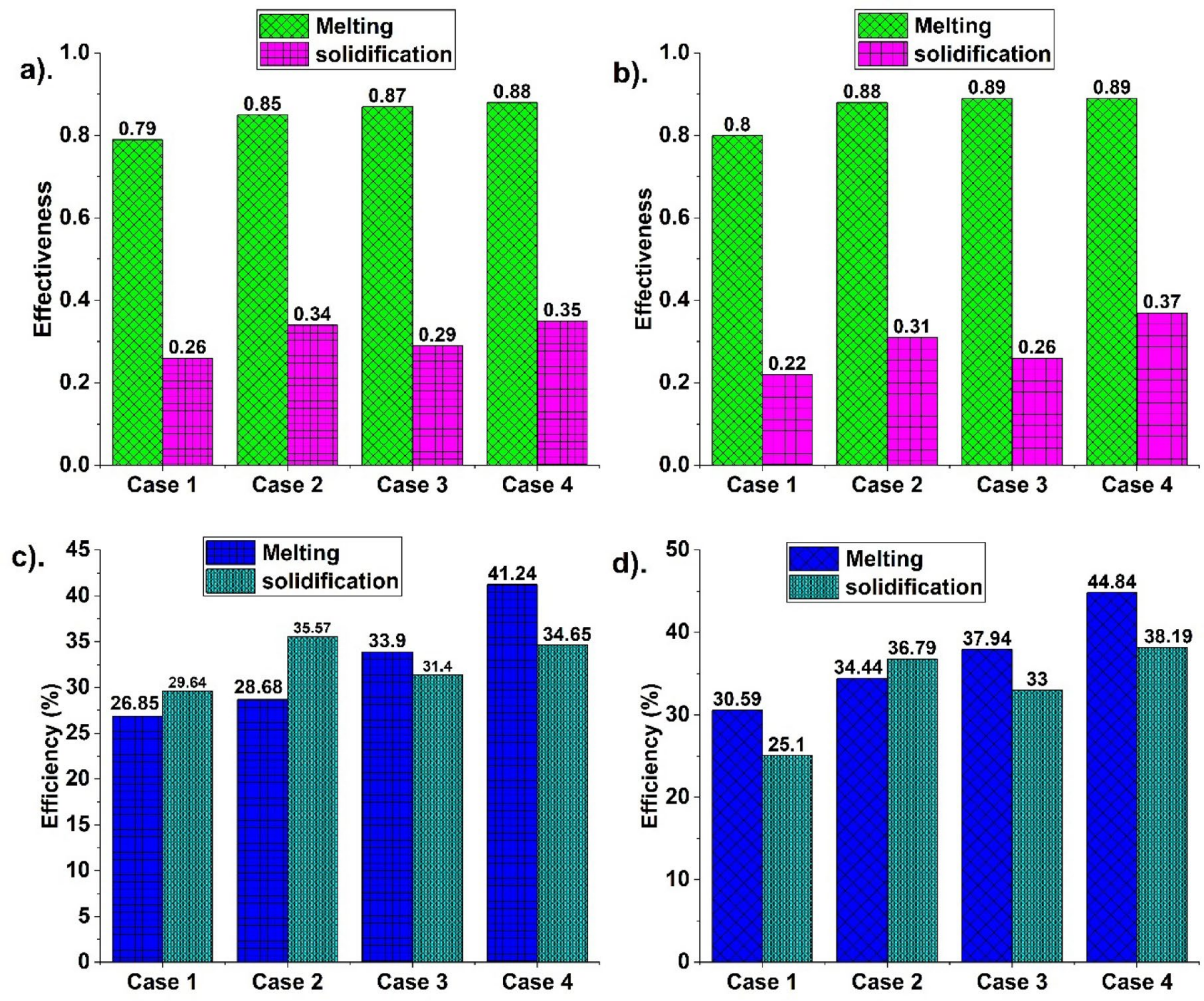
Indicate effectiveness at (**a**) 70 °C, and (**b**) 75 °C, and efficiency at (**a**) 70 °C, and (**b**) 75 °C HTF temperature for different cases.

The efficiency of TES in enhancing PCM fusion is analysed for four cases (cases 1–4). Efficiency indicates the enhanced heat transfer in expediting PCM fusion. It is the heat stored to supply. Case 1 has no fin; hence, melting is alleviated. Though it is melted from all directions, its efficiency during melting is less than in other cases. During solidification, PCM is solidified from all directions. It’s efficient in enhancing fusion, but it is comparatively less than others due to the enhanced solidification duration. Case 2 has higher efficiency than case 1 due to the fins’ incorporation. Fins positioned at the top increase storage efficiency initially. But the PCM settling alleviates melting due to the absence of fins near the bottom. During solidification in case 2, fins near the top are found to be optimal. Fin presence enhances heat transfer, thereby reducing solidification duration. Hence, the efficiency during solidification is higher in case 2. Figure 8 indicates effectiveness and efficiency for different cases (1–4) at HTF temperatures of 70 and 75 °C.

Case 3 has high efficiency during melting since fins are present near the bottom. This location is optimal in enhancing heat transfer during melting as settling is reduced. Hence, efficiency during melting is higher for case 3 than for case 2. However, it is not high during solidification due to liquid PCM accumulation. Fin absence near the top alleviates solidification, leading to less efficiency during solidification than in case 2. Case 4 is with high melting and solidification. The fins’ optimal location enhances both melting and solidification, leading to increased efficiency. Case 4 is found to have higher efficiency than the others due to optimal fin location.

The efficiency during melting and solidification is analysed. Efficiency at 70 °C during melting is 26.85, 28.68, 33.9, and 41.24%, for cases 1, 2, 3, and 4. High efficiency for case 4 indicates effective melting. Efficiency at 75 °C during melting is 30.59, 34.44, 37.94, and 44.84% for cases (1–4). High melting efficiency for case 4 indicates effective PCM melting. During solidification, the efficiencies are 25.1, 36.79, 33, and 38.19%, for 75 °C HTF temperature. High efficiency for case 4 indicates effective solidification due to enhanced heat transfer. The overall fusion is high for case 4 due to the fins’ location near the top and bottom. Its presence and penetration inside PCM enhance fusion. The fins’ location results in high conductive heat transfer. It, in turn, results in enhanced natural convection for effective melting.

Effectiveness is the heat stored/released to its maximum. Case 1 is found to be less effective due to less heat transport. Fin absence reduces heat transport for fusion, though the container is surrounded by HTF. Case 2 is with more effectiveness than case 1 due to more heat exchange. The fins’ presence enhances heat transport for increased fusion. Case 2 is with fins near the top, optimal for solidification. Hence effectiveness during solidification is higher than in case 1. However, it is less effective during melting due to the fin location. Further case 3 is analysed. Case 3 with bottom fins is optimal for melting enhancement. The fins’ presence enhances melting due to effective heat transport. Hence, case 3 is with more effectiveness than case 2 during melting. However, effectiveness during solidification is less for case 3 as fins’ location is non-optimal. Case 4 is analysed to study the effectiveness during fusion. The presence of fins throughout the container enhances the fusion process. Hence, effectiveness is higher for case 4 during fusion than others.

From Fig. 8, effectiveness is 0.79, 0.85, 0.87, and 0.88 during melting for cases 1, 2, 3, and 4 at 70 °C HTF temperature. Effectiveness at 75 °C is 0.8, 0.88, 0.88, and 0.89 for cases 1, 2, 3, and 4, indicating a high value for case 4. Effective PCM melting due to heat exchange led to more effectiveness in case 4. 75 °C is more effective due to more heat transfer and melting expedition. During solidification, effectiveness at 70 °C is 0.28, 0.34, 0.29, and 0.35 for cases 1, 2, 3, and 4. At 75 °C, the effectiveness is 0.22, 0.31, 0.26, and 0.37 for cases 1, 2, 3, and 4. More effectiveness is for case 4, indicating effective heat transfer for more fusion.

**Fig. 9:**
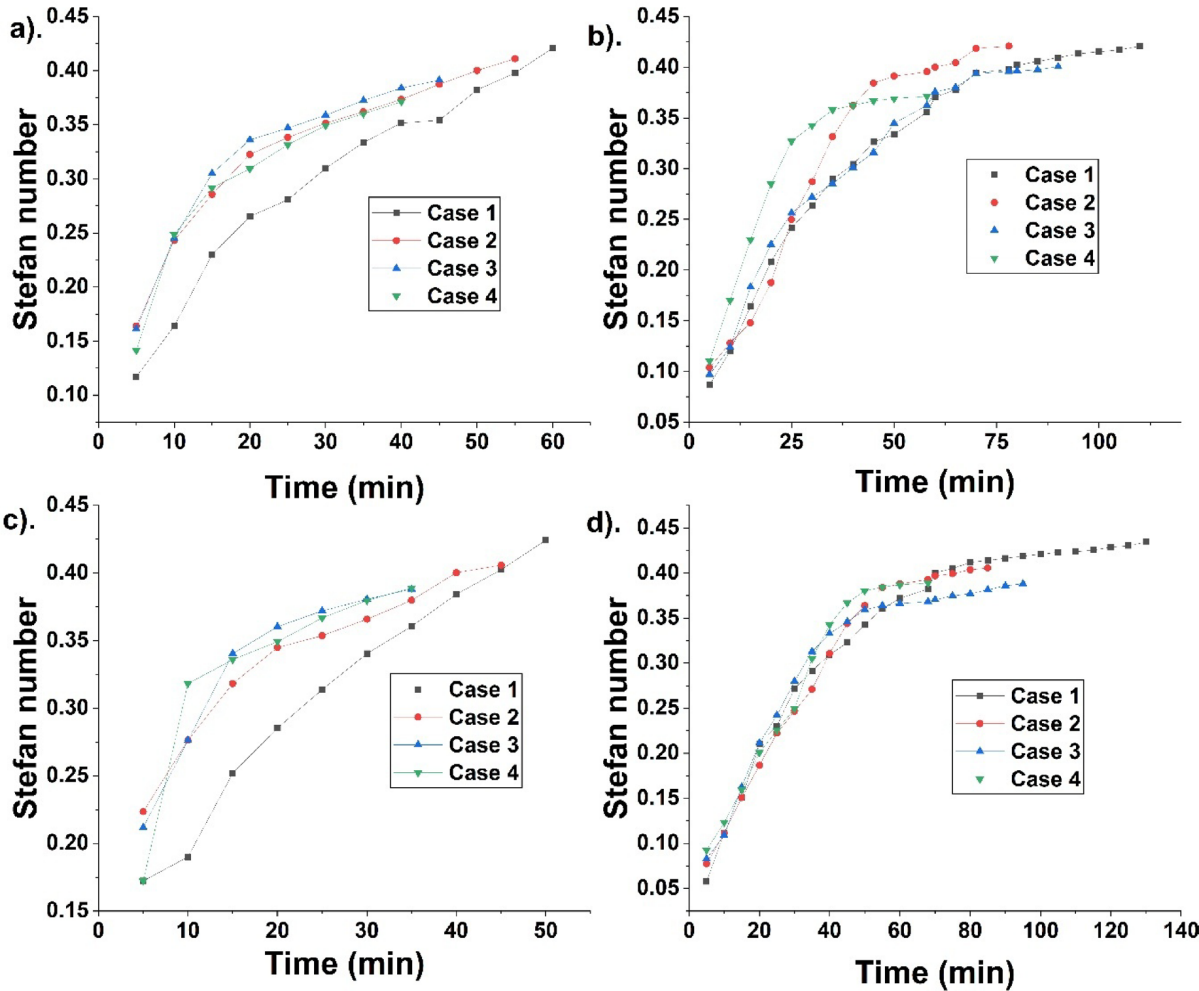
Indicate Stefan number at 70 °C during (**a**) melting and (**b**) solidification, and at 75 °C during (**c**) melting and (**d**) solidification for different cases.

Stefan number is the sensible to latent heat. A high Stefan number indicates more melting, leading to more sensible heat addition. Though there is an increase in latent heat, the sensible heating is done continuously, hence the Stefan number keeps increasing. Its value reaches a high after complete melting. Figure 9 indicates Stefan number for different cases during fusion. Stefan number at 70 °C HTF temperature is 0.42, 0.41, 0.39, and 0.37, for cases 1, 2, 3, and 4. It indicates high sensible heat addition for case 1 due to melting alleviation. Stefan number at 75 °C is 0.42, 0.4, 0.38, and 0.38, for cases 1, 2, 3, and 4. High value is found for case 1 due to melting alleviation and sensible heat addition. During solidification, Stefan number is 0.42, 0.42, 0.4, and 0.37, for cases 1, 2, 3, and 4. For 75 °C, Stefan number is 0.43, 0.4, 0.38, and 0.38, for cases 1, 2, 3, and 4. It indicates case 4 is more effective than others due to effective fusion.

### Comparative analysis of the study

A comparative analysis of all the cases is made to find the optimal fins location in enhancing heat exchange. Table 4 indicates a comparative analysis of the study. The table indicates that case 1 has a melting duration of 60 min and a solidification duration of 110 min at 70 °C. For 75 °C, the duration is 50 and 130 min for melting and solidification. Case 4 is with a melting and solidification duration of 40 and 58 min for 70 °C, while for 75 °C it is 33 and 68 min. The overall fusion duration for case 4 is 98 and 101 min, which is less than other cases. It indicates case 4 is effective in enhancing fusion, hence could be suggested for many applications in solar. The optimal fin location enhances heat transfer, thereby enhancing fusion rate, conductive heating and convective heat transfer. A melting time reduction of 10, 24, and 34% is found in cases 2, 3, and 4 compared with case 1 at 75 °C HTF temperature. A solidification time reduction of 34.6, 26.9, and 47.7% is found for cases 2, 3, and 4 compared to case 1, indicating case 4 is effective during PCM fusion.

**Table 4:** Indicates comparative analysis of the study.

Cases	Melting duration (min)		Solidification duration (min)		Overall fusion duration (min)	
	70 °C	75 °C	70 °C	75 °C	70 °C	75 °C
Case 1	60	50	110	130	170	180
Case 2	56	45	78	85	134	130
Case 3	45	38	90	95	135	133
Case 4	40	33	58	68	98	101

### Comparison with the existing study

An analysis is carried out by comparing the present study with the existing literature. Table 5 indicates a comparison with the existing study. From studies, Nanoparticles reduce fusion time from 19.16 to 5.83 min^13^. Fins reduce melting time by 63%^14^. Porous fins effectively reduce melting time by 21%, enhancing storage by 26.6%^19^. Hollow fins are effective in enhancing heat transfer^35^. Fins’ height influences melt time^36^. The studies reveal that fins’ inclusion enhances heat transport for fusion. Further, the present study with fins reduces the melting and solidification time by 34 and 47.7% compared with the finless case. The enhanced fusion in case 4 indicates that it could be suggested for application in solar flat plate collectors, where the energy is highly intermittent.

**Table 5:** Indicates comparison with the existing literature.

Reference	Outcome
Hajizadeh et al.^22^	Nanoparticle inclusion reduced fusion time from 19.16 to 5.83 min. The reduction in solidification time was 70.06%
Kok et al.^23^	Melt time reduction of 63% is achieved using fins
Jaberi and Hossainpour^28^	Melt time reduction of 21% with enhancement in storage energy of 26. 6% is achieved using porous fins
Patel et al.^35^	Capsule size reduction of 27% reduced the melt and solidification time by 12.19 and 19.17%
Meghari et al.^47^	Hollow fins reduced the melt time by 14 times than without fins
Fan et al.^48^	Fin increased height reduced the melt time by 30%
Present study	Case 4 is with a melting and solidification time reduction of 34% and 47.7%. It indicates effective fusion in case 4 than other cases

### Critical assessment and application of fins design

Case 4 is with enhanced melting and solidification characteristics. It reduces the overall fusion time; hence, it could be suggested for applications in solar systems. Solar energy is available for a shorter time; hence, the TES discussed in the study could be incorporated to enhance storage/release to mitigate intermittency. The melt and solidification time reduction of 34% and 47% for case 4 caters to the needs of real-time solar applications. It could be subjected to frequent cycling, thereby enhancing thermal output. The solidification duration reduction enables faster return to the initial state, enabling efficient thermal cycling. The results indicate that the proposed system is efficient in enhancing thermal energy storage/release. Hence, it could be suggested for applications in solar, heat recovery in industries and for compact TES systems.

## Conclusions

Solar energy is used to overcome the dependency on fossil fuels. But the high intermittency of solar energy requires an energy storage system to match supply and demand. However, energy storage systems using PCM suffer from meagre thermal conductivity, reducing heat storage/release. The use of a spherical container with fins enhances the fusion process, thereby expediting heat storage. The study uses HTF at 70 and 75 °C and a flow rate of 30 LPH to enhance the fusion process. Four cases were used for the analysis. The following conclusions were arrived at during the experiment:

- The fusion comparison indicates case 4 is optimal in enhancing PCM fusion. It displayed the melt and solidification time reduction of 34% and 47.7% when compared with case 1. The melt and solidification time reduction for case 4 is 33 min and 68 min. The melt times for cases 1, 2, and 3 at 75 °C, are 50, 45, and 38 min. The solidification durations are 130, 85, and 95 min. Further, case 4 is effective due to optimal fin location.
- Though there is a marginal variation in heat stored due to the same PCM mass, the melting and solidification efficiency at 75 °C for case and 4, was 44.84%, and 38.19%. It indicates that case 4 effectively enhances PCM fusion, hence its efficiency is high.
- The effectiveness at 75 °C for case 4 during melting and solidification was 0.89 and 0.37, indicating case 4 effectively enhances heat transfer for expedited fusion.

The analysis has contributed to the identification of a simple but effective design of locating fins at the top and bottom of a spherical container without compromising storage. It provides a guideline in the design of TES in enhancing storage, especially in solar applications where a swift storage is required due to limited irradiation. Further study on fin geometry, like length, thickness, inclination and material, could be carried out for applications involving packed bed TES systems.

## Data Availability

The datasets used and/or analyzed during the current study are available from the corresponding author upon reasonable request.

## Abbreviations

HTF: Heat transfer fluid
PCM: Phase change material
TES: Thermal energy storage

## List of symbols

$\:\dot{m}$: Mass flow rate (kg. sec^−^^1^)
C_pl_: Liquid PCM specific heat capacity (kJ. kg^−^^1^. K^−^^1^)
C_ps_: Solid PCM specific heat capacity (kJ. kg^−^^1^. K^−^^1^)
C_pw_: Water specific heat capacity (kJ. kg^−^^1^. K^−^^1^)
F_i_: Melt fraction
K: PCM thermal conductivity (W. m^−^^1^. K^−^^1^)
L: Latent heat (kJ. kg^−^^1^)
m: Mass (kg)
Q_in_: Heat supplied (kW)
t: Time (sec)
T_ini_: PCM initial temperature (K)
T_l_: Liquidus temperature (K)
T_pcm_: PCM temperature (K)
T_s_: Solidus temperature (K)
T_wi_: HTF inlet temperature (K)
T_wo_: HTF outlet temperature (K)

## Greek symbols

$$\eta$$: Efficiency
ϵ: Effectiveness
